# Exploiting the Biorefinery Potential of Spent Mushroom Substrate: The Time to Do It Is Now

**DOI:** 10.3390/molecules30234518

**Published:** 2025-11-22

**Authors:** Carlos Martín, Shaojun Xiong, Georgios I. Zervakis

**Affiliations:** 1 Department of Biotechnology, Inland Norway University of Applied Sciences, N-2317 Hamar, Norway; 2 Department of Chemistry, Umeå University, SE-901 87 Umeå, Sweden; 3 Department of Forest Biomaterials and Technology, Swedish University of Agricultural Sciences, SE-901 83 Umeå, Sweden; shaojun.xiong@slu.se; 4 Laboratory of General and Agricultural Microbiology, Department of Crop Science, Agricultural University of Athens, 11855 Athens, Greece; zervakis@aua.gr

**Keywords:** spent mushroom substrate, biorefinery, circular bioeconomy, lignocellulosic biomass, bioactive compounds

## Abstract

Spent mushroom substrate (SMS), the residual material left after mushroom cultivation, represents an abundant yet underutilized bioresource. With global mushroom production generating millions of tons of SMS annually, its disposal constitutes a missed opportunity within the circular bioeconomy. This Opinion article highlights why SMS should be repositioned as a valuable raw material for sustainable biorefineries and outlines the technological, economic, and regulatory steps needed to unlock its potential.

## 1. Background

Spent mushroom substrate (SMS) is the growing medium leftover after mushroom cultivation and harvesting. It is estimated that the production of one ton of mushrooms generates up to five tons of SMS, resulting in a global annual output of roughly 250 million tons from the mushroom industry [1]. Based on FAO data on global mushroom production and on the typical SMS-to-mushroom ratio, it can be estimated that over 200 million metric tons of SMS are produced annually in China, the world’s largest mushroom producer [2]. The next largest producers are Japan, India, and the USA with an estimated generation of 2.3, 1.6, and 1.5 million metric tons, respectively. Regarding the EU, roughly 6 million metric tons of SMS are generated each year, with Poland, the Netherlands, and Spain as the leading producers with ca. 1.2, 1.0, and 0.8 million metric tons, respectively. Most of the generated SMS remains underutilized. As global market demands for edible mushroom increase, SMS management is becoming increasingly problematic, especially in high-production regions, contributing to rising logistical and environmental concerns. However, we believe that SMS should not be considered as waste, but rather as a valuable bioresource [3]. It contains a unique combination of polymers, bioactive compounds of both fungal and plant origin, and enzymes. This particular composition distinguishes SMS from other biomass residues and positions it as a promising feedstock for biorefineries. A biorefinery is an overall concept of a processing plant where biomass feedstocks are converted into a spectrum of valuable products, such as biobased fuels, chemicals, and materials [4]. In addition to these products, a biorefinery processing SMS can also extract bioactive compounds suitable as functional ingredients for pharmaceutical, nutraceutical, and biomedical applications, provided the purification processes meet the required standards.

The valorization of agricultural and agro-industrial residues within a circular biobased economy model is a major focus of scientific interest. However, despite its potential, SMS is typically excluded from biomass inventories and not prioritized in strategic research programs. This exclusion highlights that neither academia nor industry has yet fully recognized the true potential of SMS, which is a concerning oversight. Hereby, we argue that it is time to reassess the role of SMS as a promising raw material for the sustainable production of valuable biobased products in biorefineries.

## 2. What Is So Unique About Spent Mushroom Substrate (SMS)?

Although SMS is to a large extent composed of biomass derived from agricultural or forest residues, it is qualitatively different from typical lignocellulosic materials. Due to its dual biological origin, SMS contains not only residual plant components but also a substantial amount of fungal biomass, resulting from mycelium growth on the cultivation substrate. During mushroom cultivation, fungi partially degrade lignin and polysaccharides, leaving behind the non-consumed lignocellulosic fraction along with mycelium and other fungal-derived material (including secondary metabolites and other bioactive compounds). SMS of white-rot fungi, which constitute a major share of cultivated mushrooms, typically contains less lignin and more extractives than other lignocellulosic materials, as exemplified by comparing sugarcane bagasse (a gramineous bioresource), maritime pine (softwood), and eucalyptus (hardwood) on one side, and SMS of shiitake (*Lentinula edodes*) and oyster mushrooms (*Pleurotus* spp.), on the other side (Table 1).

The partial degradation of lignin during mushroom cultivation increases the accessibility to hydrolyzing enzymes of the cellulose remaining in the spent substrate, as compared to that in the initial substrate [9]. This is a significant advantage from a biorefinery perspective, as it facilitates biomass deconstruction without the need for conventional pretreatment, which would otherwise increase costs and generate inhibitors of enzymes and microorganisms. Additionally, fungal β-glucans, chitin, ergosterol, proteins, and secondary metabolites are retained in the SMS. Many of these fungal-derived compounds are known to exhibit antioxidant, antimicrobial, or immunomodulatory properties, among other bioactivities [10], which are not to be found in common lignocellulosic biomass.

Hence, SMS can be considered a multifunctional bioresource, enriched with hydrolyzable cellulose and bioactive compounds. This makes it suitable as a source of fermentable sugars for producing biofuels and biomaterials, and of extracts for producing high-value-added products to be used in the nutraceutical, functional food, or cosmetic sectors.

## 3. Unrealized Potential and Missed Opportunities in SMS Exploitation

Despite its high potential value, SMS is generally downgraded to low-value uses (usually as soil amendment after composting) [11], while it is also disposed of as waste by incineration, burial, and piling [12]. Although existing applications contribute to organic matter recycling, the full potential of SMS bioactive compounds and structural polymers is far from being exploited. Mushroom production plants generally lack the facilities required for applying systematic valorization strategies to SMS. As a rule, the infrastructure and technical insight needed to integrate SMS into biorefinery pipelines are far beyond the scope of today’s mushroom industry.

This represents a missed opportunity for several economic sectors. For example, the emerging lignocellulosic biorefinery sector, which is interested in low-cost feedstocks, would benefit from the recognition of SMS as a prioritized raw material for producing biofuels and platform chemicals. Furthermore, the pharmaceutical, cosmetic, and food industries could benefit from the SMS potential as a natural source of antioxidants and other bioactive compounds, provided that its use is promoted by national policies.

Although some lab-scale research has demonstrated the suitability of SMS for bioconversion through saccharification and fermentation [13], extraction of bioactive compounds [14], and thermochemical conversion [15], the results rarely progress beyond the laboratory bench. This fact indicates that SMS remains underutilized not because of limited scientific interest, but because of inadequate institutional support to establish it as a mainstream feedstock. Therefore, the existing gap hinders the translation of scientific knowledge into scalable, innovative processes of interest for investors and entrepreneurs.

## 4. The Biorefinery Potential of Spent Mushroom Substrate (SMS)

### 4.1. What Does SMS Offer to Biorefineries?

In lignocellulosic biorefineries, cellulose, hemicelluloses, and lignin are transformed into smaller molecules, which are then valorized into biofuels, chemicals, or other materials. During mushroom production, the substrate polymers are partially degraded, but a substantial portion of them remains in the SMS after cultivation. This remaining fraction of carbohydrates and lignin can be further utilized in biorefinery processes.

Cellulose saccharification is a key step in biorefineries. Enzymatic saccharification requires pretreatment to reduce the interference of lignin and hemicelluloses, thereby enhancing enzyme accessibility. Compared to conventional lignocellulosic materials, SMS exhibits higher cellulose digestibility, as much of the lignin and hemicellulose interference has already been reduced during fungal growth. Therefore, when SMS is used as feedstock, the need for energy- and material-intensive pretreatment is minimized. For example, in studies with shiitake SMS, we have achieved cellulose conversions over 80% (*w*/*w*), demonstrating its high enzymatic digestibility [8,9]. SMS enzymatic saccharification results in sugar-rich hydrolysates, which are suitable for fermentation, not only because they provide sugars as a carbon source, but also because they are free from inhibitors typically formed during thermochemical pretreatments, and are rich in nutrients that support microbial growth [13].

SMS is also a source of valuable polymers, such as lignin, chitin, and crystalline cellulose, which can be recovered by chemical processes following biorefinery routes. These polymers can be used to formulate composites, nanoparticles, and other advanced materials [16] with the potential to replace fossil-based products.

Unlike other lignocellulosic residues, SMS offers the added value of bioactive compounds, which are either structural components of the mycelium, products of the fungus’ metabolism, phytochemicals from the original substrate, or oligomers formed by partial lignocellulose degradation during cultivation. SMS bioactive compounds can be recovered using green extraction methods and directed toward value-added applications.

### 4.2. Integrated Processing

**Figure 1:**
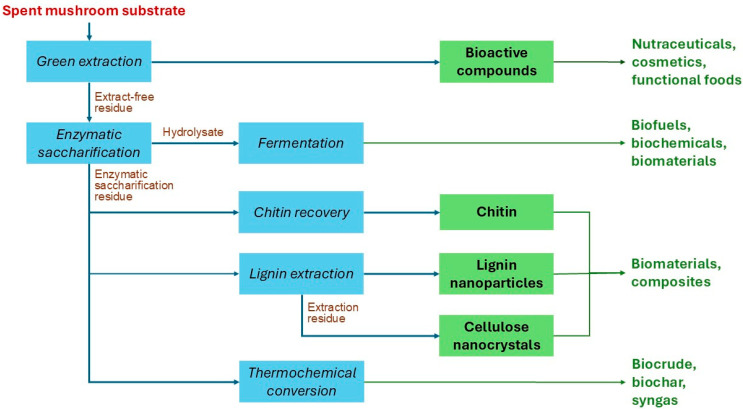
Simplified flow diagram of a biorefinery using spent mushroom substrate as feedstock. Blue boxes represent processes, and green boxes represent value-enabling intermediates. Brown regular text denotes process streams, and green bold text denotes products.

A simplified flow diagram of our envisioned biorefinery is shown in Figure 1. The process includes, as the first step, the extraction of bioactive compounds, which can serve as precursors of high-value products, such as nutraceuticals, cosmetics, or functional foods. The extraction also results in an extract-free residue, which represents approximately 70% or 80% of the initial dry weight of the SMS of shiitake or oyster mushrooms, respectively [14].

The extract-free residue (EFR) undergoes saccharification to produce a sugar-rich hydrolysate. Enzymatic conversion of over 80% (*w*/*w*) of the cellulose and hemicelluloses can be anticipated in the case of shiitake’s SMS [8]. The hydrolysate, typically containing approximately 30–40 g/L glucose and 10–15 g/L xylose, can then be fermented into biofuels, biopolymers, or other products (Figure 1). A residual solid stream, hereafter referred to as enzymatic saccharification residue (ESR), is also formed during saccharification. The ESR yield is expected to be approximately 35% of the weight of the EFR subjected to saccharification, which corresponds to around 25% of the initial SMS dry weight. ESR contains up to 35% (*w*/*w*) lignin, 20–25% cellulose, and some amount of chitin [8]. ESR cellulose is expected to exhibit high crystallinity, given that most of the amorphous cellulose has been removed during enzymatic saccharification. Although data on chitin content in SMS are scarce, our preliminary findings indicate values ranging from 5 to 30 mg/g SMS (Xiong et al., unpublished material). The saccharification residue can be used to recover either chitin or lignin nanoparticles (LNP), while the residue from lignin extraction can subsequently be processed into cellulose nanocrystals (CNC). Chitin, LNP, and CNC can be applied in the formulation of innovative biobased materials. Alternatively, the saccharification residue, as well as any other remaining residue, can be subjected to thermochemical conversion to biocrude, biochar, or syngas, which can serve as renewable fuels or chemical precursors, contributing to closing the carbon loop [15]. This integrated strategy maximizes biomass utilization and supports zero-waste objectives in line with the principles of a circular bioeconomy.

We have partially demonstrated sections of this route by extracting bioactive compounds from shiitake and oyster mushroom SMS [14], running enzymatic saccharification after the extraction of bioactives [17], fermenting the hydrolysates to ethanol [13] and exopolysaccharides [18], and recovering lignin from the saccharification residues [8]. The results of our experiments, together with the insights and outcomes from conceptual studies on specific conversion routes [19], demonstrate that SMS is a versatile and modular feedstock whose various fractions can be selectively recovered and processed through different pathways, depending on the desired product(s) portfolio. Repositioning SMS within the framework of biorefineries enables multi-product valorization according to the cascading concept, whereby extraction, bioconversion, and recycling steps are integrated to maximize both sustainability and economic viability.

## 5. The Bottlenecks

Despite its promising potential, the widespread adoption of SMS as a biorefinery feedstock remains hindered by several barriers, as discussed below.

### 5.1. Compositional Variability

One of the key challenges in valorizing SMS is its variable composition, which can change widely depending on the cultivated mushroom species, the initial growth substrate, and the cultivation conditions. The compositional variability of SMS can be illustrated by the wide ranges in the content of cellulose, hemicelluloses, lignin, and extractive compounds reported for shiitake and oyster mushroom SMS (Table 2). Even within the same species, use of different strains and/or small modifications to the substrate recipe can induce metabolic changes, altering carbohydrate and lignin content and bioactive compound profiles [20]. Conversely, deliberate adjustment of substrate composition could be used to enhance the bioactive content of residual fungal biomass, adding value for downstream applications [21,22]. However, this intrinsic compositional variability still hinders the use of SMS in biorefineries, leading to unpredictable yields and product quality. The absence of standardized characterization protocols further complicates SMS integration into industrial value chains. Addressing this bottleneck requires the development of reliable methods for SMS analysis to ensure reproducibility and compatibility with industrial operations.

### 5.2. Logistical Limitations

The high moisture content of freshly produced SMS presents a major challenge for its transport to industrial facilities. The water content of the initial substrate is typically around 60%. However, controlled irrigation during cultivation, water release from fungal metabolism, condensation of water vapor, and increased water retention capacity -as polymers degrade and the substrate becomes more porous- can raise SMS water content to a level (around 80%) that complicates handling and promotes microbial spoilage.

Furthermore, SMS has a low bulk density, increasing transportation costs. Although dewatering SMS can lower costs, it is costly itself and requires careful evaluation of the adverse effects it might have on efficiency, transportation savings, and SMS quality.

Another logistical challenge is that SMS is often produced in decentralized locations, far from biorefinery facilities. This limits its transportability and shelf life, particularly for bioactive compound extraction, whereby rapid degradation occurring after harvest can compromise extract suitability for further processing into high-value outputs.

Some potential measures to address the logistical challenges include implementing low-cost dewatering and densifying strategies to improve transportability, using mobile extraction units to preserve bioactive compounds, and establishing local biorefinery hubs to improve shelf life and facilitate processing. Additionally, fostering farmer cooperatives could streamline collection and further reduce costs.

### 5.3. Lack of Processing Infrastructure

Lignocellulosic biorefining is still an emerging industry, and most current plants are pioneering facilities that are equipped to process specific feedstocks, such as certain agricultural or forestry residues, while SMS is so far outside their scope. Adapting existing infrastructure to handle SMS, with its unique composition, moisture content, density, and preprocessing demands, requires substantial investment to implement the necessary technical modifications. Looking forward, we believe that as the market evolves and technologies mature, biorefineries will become more versatile, allowing SMS to be integrated into their feedstock portfolio and play a key role in the global bioeconomy.

### 5.4. Limited Research

Although SMS has been the focus of some biorefinery-related research, the number of reported studies is considerably lower than that on other biomass materials, such as corn stover, sugarcane bagasse, wheat straw, or forest residues. The lack of comprehensive studies on SMS hinders the development of optimized valorization processes. While early studies have highlighted SMS’s potential as a valuable resource, many critical aspects, such as the identification of pre-processing methods, elucidation of microbial degradation pathways, and implementation of cost-effective recovery strategies, remain poorly understood. This research gap prevents developing technologies that could streamline SMS utilization in biorefineries. Consequently, limited research slows down industrial adoption, making it harder for companies to justify the investment needed on a commercial scale.

### 5.5. Public Perception

Despite the potential of SMS as a valuable bioresource, its categorization as a waste or a compostable by-product remains a major barrier to its broader acceptance. This outdated perception and the general public’s limited awareness undermine the perceived economic value of SMS and prevent its recognition as a strategic feedstock. As a consequence, research investment is discouraged, hindering the development of innovative solutions. We believe that a coordinated effort is required to reframe SMS as a value-enabling resource and change the public narrative surrounding it. Without such an effort, the integration of SMS into biorefinery processes will continue to be delayed.

### 5.6. Policy and Regulatory Gaps

The perception of SMS as an inconsistent bioresource due to the technical limitations discussed in Section 5.1, Section 5.2 and Section 5.3 and the underestimation of its true raw material value have discouraged its inclusion in national biomass strategies and policies. For instance, SMS is not explicitly mentioned among the biomass residues eligible for valorization in Poland’s Renewable Energy Sources Act [27], the Dutch Climate Agreement [28], or the Spanish Circular Economy Strategy [29], which are the national biomass valorization frameworks of the three largest mushroom-producing countries in Europe. As a consequence, SMS is not included in bioeconomy roadmaps or funding calls. This regulatory ambiguity limits the financial resources available to support SMS research, restricts industrial innovation, and creates uncertainty for investors and entrepreneurs.

### 5.7. Economic Barriers

The economic feasibility of SMS valorization depends on the cost structure of mushroom production. In Europe, high production costs result in elevated mushroom prices, limiting market growth and, consequently, the availability of SMS for large-scale use. These costs stem mainly from labor- and energy-intensive cultivation methods, and outdated technologies requiring upgrading. Moreover, limited consumer demand in some areas, where acceptance of cultivated mushrooms remains low, reduces incentives for producers and investors. Lowering cultivation costs and increasing consumption would help expand both mushroom production and SMS valorization potential.

## 6. Why Now Is the Time

Overcoming the above-mentioned bottlenecks requires coordinated efforts between researchers, mushroom producers, biorefinery engineers, policymakers, and funding agencies. These efforts cannot be postponed, as converging trends in policy, sustainability, and innovation only underscore their urgency.

### 6.1. Circular Bioeconomy Goals and Agricultural Waste Valorization

The EU Green Deal [30], the UN Sustainable Development Goals [31], and national sustainability agendas worldwide emphasize the need to accelerate the transition to a circular bioeconomy. Diversifying biomass feedstocks is a critical step toward that goal. SMS, being renewable, underutilized, and available at scale, aligns well with this vision. Furthermore, valorizing SMS contributes to the sustainability of waste management systems and supports the strategies of governments and environmental agencies in developing waste-to-resource solutions to achieve circular bioeconomy goals.

### 6.2. Technological Readiness

Innovative techniques in green extraction, pretreatment, saccharification, and fermentation provide solutions to recover valuable intermediates from SMS, amplifying its potential as a biorefinery feedstock. Methods such as supercritical CO_2_ extraction and microwave-assisted extraction allow for the efficient and sustainable recovery of bioactive compounds. Similarly, recent advances in pretreatment, saccharification, and fermentation processes—demonstrated at both pilot and commercial scales in 2G ethanol biorefineries—improve the conversion of complex biomass into biobased products. These developments improve the prospects for scalable implementation of SMS integration into sustainable bioeconomy models.

### 6.3. Emerging Markets for Bio-Based Products

The rising demand for natural antioxidants, prebiotics, and sustainable materials is transforming the food, cosmetic, and pharmaceutical industries, while also driving innovation in functional packaging. Consumers increasingly seek products of non-animal origin that align with ethical, health-conscious, and sustainability-driven values. Natural antioxidants are in high demand for their skin protective, anti-aging, and wellness benefits, while prebiotics are gaining popularity for their digestive health benefits. At the same time, biobased biodegradable packaging offers sustainable alternatives to conventional materials. These trends underscore the SMS potential as a promising feedstock for biorefineries. Rich in bioactive compounds, SMS can be converted into high-value antioxidants, prebiotics, and biopolymers, offering vegan-friendly, sustainable solutions that respond to the growing demand for ethical, eco-conscious, and health-oriented products across these emerging markets.

### 6.4. Cross-Sector Collaboration Potential

Research on a hypothetical SMS-based biorefinery has great potential to serve as a ‘binder’ for interdisciplinary collaboration, including projects funded by large research and innovation framework programs such as Horizon Europe. This is because SMS integrates elements from agriculture, biotechnology, and bioenergy, while also offering significant potential for high-value applications in sectors at the interface of food, wellness, pharmaceuticals, and functional materials. Early investment in public–private partnerships could be a visionary step toward launching interdisciplinary innovation across the entire SMS biorefinery value chain.

## 7. Conclusions

In summary, the scientific rationale is strong, the environmental urgency is clear, and the technological tools are increasingly ready. What is needed now is a strategic commitment to unlock the full potential of spent mushroom substrate (SMS).

This is the moment for coordinated action by researchers, industry, and policymakers to remove existing barriers and bring SMS-based biorefineries to life. Researchers must focus on optimizing scalable processes and fostering interdisciplinary collaboration. Industry players should invest in flexible infrastructure, develop innovative logistics, and strengthen value chain partnerships to ensure consistent feedstock supply and market development. Meanwhile, policymakers need to integrate SMS into circular bioeconomy frameworks, offer targeted incentives and funding mechanisms, and raise public awareness to improve perception and regulatory clarity. With the right support, SMS can become a key component of future sustainable biorefineries.

## Figures and Tables

**Table 1:** Summary of the composition of different types of lignocellulosic biomass, including spent mushroom substrate (SMS). The content of each component is given in % (*w*/*w*).

Biomass Material	Cellulose	Hemicelluloses	Lignin	Extractives	Source
Sugarcane bagasse	36.5	21.3	19.1	4.9	[5]
Maritime pine	45.0	22.2	26.8	2.9	[6]
Eucalyptus	42.0	22.2	22.9	4.7	[7]
Shiitake SMS	28.1	12.8	6.6	25.1	[8]
Oyster mushroom SMS	30.6	18.6	12.7	13.0	[8]

**Table 2:** Compositional range of the main components of spent mushroom substrate of shiitake (*Lentinula edodes*) and oyster (*Pleurotus* spp.) mushrooms as reported by different sources. The content of each component is given in % (*w*/*w*).

Fungal Species	Cellulose	Hemicelluloses	Lignin	Extractives	Source
*Lentinula edodes*	24.8–44.3	7.6–17.2	6.6–26.1	22.3–30.0	[8,9,14,23,24,25,26]
*Pleurotus* spp. ^1^	27.3–48.7	8.8–22.1	11.0–23.0	13.0–22.6	[8,14,17,25]

^1^ Data on *P. ostreatus*, *P. eryngii*, *P. pulmonarius*, and hybrid *P. ostreatus* × *P. eryngii* are compiled.

## Data Availability

No new data were created or analyzed in this study. Data sharing is not applicable.
